# SARS‐CoV‐2 Infection With Alpha B.1.1.7 Virus Induced Higher Antibody Responses Than Earlier Non‐VOC Variants During the First Waves of the COVID‐19 Pandemic in Norway

**DOI:** 10.1111/apm.70102

**Published:** 2025-12-10

**Authors:** Gro Tunheim, Marta Baranowska‐Hustad, Fridtjof Lund‐Johansen, Even Fossum, Sabin Bhandari, Liva Kukule, Thea Kristine Rogne Møller, Elisabeth L. Vikse, Terese Bekkevold, Fredrik Oftung, Anna Hayman Robertson, Lisbeth M. Næss

**Affiliations:** ^1^ Division of Infection Control Norwegian Institute of Public Health Oslo Norway; ^2^ Department of Immunology Oslo University Hospital and University of Oslo Oslo Norway

**Keywords:** Alpha, COVID‐19, IgG antibodies, non‐VOC, SARS‐CoV‐2, severity, symptoms, viral load

## Abstract

Antibody levels induced by SARS‐CoV‐2 infection have been reported to be associated with specific symptoms, disease severity, and viral load. In this study we investigated whether antibody responses were associated with virus type (Alpha B.1.1.7 or non‐variants of concern (non‐VOC)), viral load and clinical outcome in unvaccinated non‐hospitalized adults with PCR‐confirmed SARS‐CoV‐2 infection. Serum samples, questionnaires and symptom diaries were collected longitudinally (Day 0–180) between May 2020 and June 2021. IgG levels against ancestral Wuhan antigens and antibodies inhibiting RBD‐ACE2 interaction were measured by multiplex immunoassay and flowcytometry, respectively. Antibody neutralization assays were performed with B.1 and B.1.1.7 viruses. Viral load was measured by digital‐droplet PCR, and virus isolates were sequenced. Factors influencing IgG levels were investigated using Bayesian multilevel models. Alpha‐cases had 2.6–3.2‐fold higher IgG levels against RBD, nucleocapsid, and spike on Day 14, and higher antibody‐mediated inhibition of ACE2‐RBD interaction compared to non‐VOC cases. Alpha‐cases displayed 1.8‐ and 5.4‐fold higher neutralizing antibody titers than non‐VOC cases against B.1 and B1.1.7, respectively, but both non‐VOC and Alpha cases displayed the lowest ratio of binding to neutralizing antibodies against their infecting virus type. Alpha cases reported more symptoms than non‐VOC cases, but the severity of disease was similar. Nausea was significantly associated with higher IgG levels, while no association was found for viral load, despite Alpha cases having higher viral loads than non‐VOC cases. This study shows higher antibody responses induced by the more transmissible Alpha virus compared to earlier non‐VOC variants after mild SARS‐CoV‐2 infection. Reporting of nausea was positively associated with IgG levels.

## 1 Introduction

SARS‐CoV‐2 infection induces antibodies against the viral surface spike (S) protein and the internal nucleocapsid (N) protein [1]. The spike protein consists of subunits where subunit S1 contains the receptor binding domain (RBD) responsible for binding to the host angiotensin‐converting enzyme‐2 (ACE2) receptor. Antibodies targeting S1 and RBD can therefore have neutralization activity [1]. Although a laboratory‐based correlate of protection against SARS‐CoV‐2 infection has not been precisely defined, both neutralizing and binding antibodies against spike protein have been shown to be of importance [2].

Since the emergence of the ancestral Wuhan virus in 2019, several variants have emerged due to viral mutations. Such variants may have increased transmission, lead to more severe disease, or reduce the effectiveness of available vaccines. Consequently, these viruses were termed variants of concern (VOC) by the World Health Organization (WHO) [3]. The first VOC, the Alpha variant (Pango lineage B.1.1.7) with eight mutations in the S protein [4], was detected in the UK in September 2020. In Norway, the earliest Alpha infection was reported in late November 2020 and subsequently detected in 50%–90% of weekly cases between February and July 2021 [5].

We and others have previously shown that the Alpha variant had increased transmissibility compared to the ancestral Wuhan‐type virus [6, 7]. The Alpha variant was also reported to be associated with increased numbers of hospitalizations and admissions to intensive care in Norway and other European countries compared with non‐VOC infections [5, 8]. Whether this was due to the increased transmissibility, or more severe disease is unclear, although some studies indicate the latter [9, 10, 11]. Higher viral loads have also been reported for the Alpha variant [6, 10], and viral load could possibly influence antibody responses [12, 13].

SARS‐CoV‐2 infection causes a variety of symptoms and a broad range of disease severities. Clinical outcome is influenced by virus variant, age, male sex and several underlying conditions [9, 14, 15, 16, 17]. Anti‐SARS‐CoV‐2 antibody levels have been shown to be higher in hospitalized patients than in non‐hospitalized cases [18, 19] and to be associated with disease severity [20, 21]. Specific symptoms such as fever and cough may also be associated with higher levels of SARS‐CoV‐2 antibodies [19, 22].

During the first waves of the pandemic in Norway, we conducted a SARS‐CoV‐2 household transmission and immune response study based on a protocol from the WHO [6, 23]. In the present study we have characterized the longitudinal antibody response in the adult participants who were closely followed up with biological sampling and monitoring of clinical status for 6 months. The shift from non‐VOC viruses to Alpha during the study period allowed us to investigate the influence of these virus variants on serum antibody responses, and whether antibody levels were associated with viral load, specific symptoms, or severity of disease. To our knowledge, a comparison between antibody responses after infection with Alpha and earlier Wuhan‐like non‐VOC strains (from here on termed non‐VOC) has not been performed. The results of this descriptive study thereby expand the knowledge about antibody responses in relation to viral strain, viral load, and clinical outcome in unvaccinated and non‐hospitalized COVID‐19 cases.

## 2 Methods

### 2.1 Study Population

**FIGURE 1:**
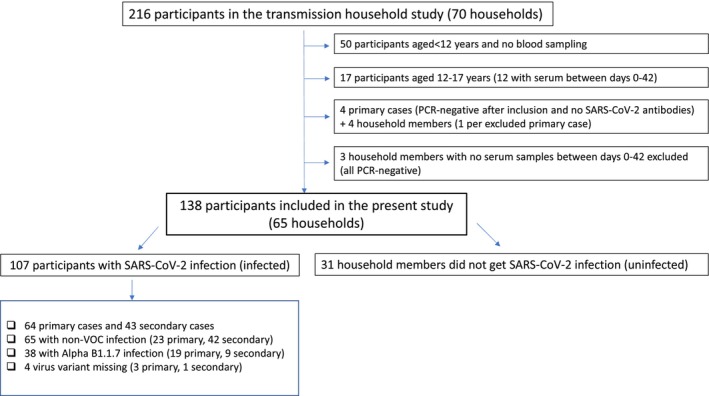
Flow chart of study participants. Infection was rRT‐PCR‐confirmed. Whole genome sequencing was used to determine the virus variant. Non‐VOC: Non‐variant‐of‐concern. Primary case: The individual who introduced the SARS‐CoV‐2 infection into the household. Secondary cases: Household members infected by a primary case.

Households with an RT‐PCR‐confirmed SARS‐CoV‐2‐infected individual, defined as the “primary case”, living with ≥ 1 other person, were recruited after mandatory municipality testing in Oslo and Viken county between May 2020 and April 2021 [6]. Household members were defined as individuals who resided with the primary case. Only participants ≥ 18 years with at least one serum sample were included in the present study (Figure 1). Eight individuals from four households were excluded as SARS‐CoV‐2 infection could not be PCR‐confirmed after inclusion, nor did they develop antibodies against SARS‐CoV‐2, suggesting that the primary cases were false positives in the municipality test setting.

A household member was considered a “secondary case” if they had a positive PCR test, and their symptom onset/PCR‐positive test (which ever came first; defined as T0, see below) was within 14 days after T0 of the primary case.

Participating households were repeatedly visited at home by a study team. Sampling days were based on the WHO COVID‐19 household study protocol [23]. The first visit was labeled Day 0 (D0), and blood samples were collected on D0, D7, D14, D28, D42 and D180 using BD Vacutainer Serum Tubes, Silica (clot activator) (Becton, Dickinson and Company, Franklin Lakes, USA). Samples were centrifuged for 15 min at 1000 × *g*, and sera were stored at −20°C.

COVID‐19 vaccines were not available until December 2020. None of the participants were vaccinated between D0 and D42, whereas 12 individuals (7 vaccinated and 5 missing vaccination status) were excluded from the D180 analyses.

### 2.2 Time Since Infection Onset

All primary and many secondary cases had tested positive prior to D0. Therefore, we also obtained information about the earliest date (T0) of either the first symptom or the first positive PCR test. From T0 and Dx (x is any visit day), we calculated time since infection onset (Tx days).

### 2.3 Quantitation of IgG by Multiplex Immunoassay (MIA)

Serum IgG levels against spike S1, RBD and N of ancestral Wuhan origin were measured using an in‐house MIA, based on a published method by den Hartog [24]; however, we switched to the RBD antigen 40,592‐408H (Sino Biological Europe GmbH, Eschborn, Germany) after personal communication with den Hartog. The detection antibody was goat anti‐human‐IgG‐PE (P8047, Sigma‐Aldrich). Anti‐SARS‐CoV‐2 immunoglobulin (21/234) (National Institute for Biological Standards and Control (NIBSC), Potters Bar, England) was used as a standard. The MIA was validated against a flow cytometric method [25] (all samples) and the Bio‐Plex Pro Human lgG SARS‐CoV‐2 Serology Assay (Bio‐Rad, Hercules, CA, USA) (subset). All sera were tested in dilutions 1:200 and 1:2000. Sera with high antibody levels were retested using higher dilutions.

### 2.4 Inhibition of ACE2‐RBD Interaction

Sera were incubated with beads coupled with Wuhan‐type RBD as previously described [25]. After centrifugation and removal of serum, digoxigenin‐labeled recombinant ACE2 was added. After incubation and washing, monoclonal anti‐digoxigenin conjugated to R‐Phycoerythrin was added. If the serum contains antibodies binding to RBD epitopes involved in the ACE2‐RBD interaction, this gives a decrease in the fluorescent signal compared to beads incubated without serum.

### 2.5 Neutralization Assays

D28 sera from individuals infected with non‐VOC or Alpha (*n* = 30 sera per virus type) were randomly selected and run in neutralization assays against SARS‐CoV‐2 strains B.1 (EPI_ISL_449791) and B.1.1.7 (EPI_ISL_1547627) using Vero E6 cells as previously described [26]. In short, serum samples were diluted 2‐fold starting at a dilution factor of 1:10 and incubated with either B.1 or B.1.1.7 for 1 h before being added onto Vero E6 cells seeded at a concentration of 1.2 × 10^4^ cells per well. Serum samples that did not give neutralization at a 1:10 dilution were given a titer of 5.

### 2.6 Virus Type and Viral Load

Oropharyngeal samples and saliva were collected on D0, D3, D7, D10, D14, D28, and D42 according to the study protocol and tested for SARS‐CoV‐2 by rRT‐PCR for all participants as previously described [6]. Virus type was determined by Next Generation Sequencing (NGS) [6]. Viral load was measured for PCR‐positive individuals using droplet digital PCR (ddPCR) in the saliva sample with the lowest Ct value (*n* = 99) [6].

### 2.7 Symptoms of SARS‐CoV‐2 Infection

All participants filled out a questionnaire on D0 adapted from the WHO protocol [23] and self‐recorded symptoms daily between D0 and D28 in a symptom diary with 12 pre‐specified symptoms. A participant was positive for a symptom if the symptom was reported at least once between D0 and D28, and within 14 days of the first positive PCR test to ensure that the symptom was related to the SARS‐CoV‐2 infection. For uninfected participants, symptoms recorded between D0 and D14 were analyzed.

### 2.8 Severity of SARS‐CoV‐2 Infection

In questionnaire Q‐DX, completed during home visits (D28/D42) or by telephone, infected participants were asked to grade their severity and duration of disease. These two variables were combined to generate three severity categories (85.0% of cases). Participants who reported being “not ill” or “barely ill” for 1–2 days, were defined as “not ill”. Participants who reported being “very ill” or “quite ill” for > 5 days were defined as “moderately ill”. The remaining participants were categorized as “mildly ill” (defined as “barely ill” for > 2 days, moderately ill independent of length of illness or “quite ill” for ≤ 5 days).

### 2.9 Statistics

Analyses were performed using STATA/SE 18.0 for Windows (StataCorp LLC, College Station, TX, USA) and R version 4.4.1 (R Core Team 2024) [27]. Graphs were made using GraphPad Prism 9 (GraphPad Software, San Diego, CA, USA). Categorical data were analyzed using a chi‐squared test. The Mann–Whitney *U* test or Kruskal–Wallis test followed by a Dunn's test was used for unpaired data and the Wilcoxon signed‐rank test was used for paired data.

To investigate factors influencing IgG levels collected longitudinally between D0 and D42 from infected participants, we utilized the brms package version 2.22.0 in R which fits complex Bayesian multilevel models using Stan [28] (one model per antibody specificity). Model details can be found in Data S1. Point estimates with 95% credible intervals (CrI) are reported.

## 3 Ethics

The study was approved by the Regional Committees for Medical and Health Research Ethics in Norway (118354). A written informed consent was obtained from all participants. The study was conducted in accordance with the Declaration of Helsinki.

## 4 Results

### 4.1 Study Population

Overall, 138 adults enrolled in the Norwegian COVID‐19 household study [6] were included (Figure 1; Table 1). Median age was 38 years (range 18–73 years); 51.5% were women and 21.7% had an underlying medical condition (Table 1). SARS‐CoV‐2 infection was PCR‐confirmed in 107 (77.5%) participants: 46.4% primary cases and 53.6% secondary cases (Table 1).

**TABLE 1:** Characteristics of the study population.

	All participants (*n* = 138)				Infected only (*n* = 107/103^c^)					*p*
	Total (*n* = 138, 100%)	Infected^a^ (*n* = 107, 77.5%)	Uninfected (*n* = 31, 22.5%)	*p*	Primary cases^b^ (*n* = 64, 59.8%)	Secondary cases^b^ (*n* = 43, 40.2%)	*p*	Non‐VOC‐infected (*n* = 65, 63.1%)	Alpha‐infected (*n* = 38, 36.9%)	
Age (years), median (IQR)	38 (29–47)	39 (31–47)	33 (26–44)	0.144	38 (31–36.5)	40 (29–49)	0.802	40 (31–50)	38 (29–47)	0.227
Females, *n* (%)	71 (51.5)	54 (50.5)	17 (54.8)	0.668	31 (48.4)	23 (53.5)	0.608	31 (47.7)	19 (50.0)	0.821
Underlying conditions^d^	30 (21.7)	25 (23.4)	5 (16.1)	0.390	13 (20.3)	12 (27.9)	0.363	16 (24.6)	7 (18.4)	0.340
Viral load (RNA copies/μL eluate), GMC (95% CI) (*n*)	n.a.	1038 (528–2043) (98)	n.a.	—	673 (272–1666) (58)	1946 (697–5432) (40)	0.130	436 (208–916) (61)	6749 (2126‐21,431) (34)	**0.0001**
Days between onset of infection and the first study visit (T0), median (IQR)	n.a.	4 (−2–9)	n.a.	—	4 (2–9)	1 (−2–5)	< **0.0001**	4 (−1 to 8)	3 (0–6)	0.207

*Note:* *P*‐values < 0.05 in bold.

Abbreviations: CI, confidence interval; GMC, geometric mean concentration; IQR, interquartile range; n.a., not applicable.

^a^ Infection was not PCR‐confirmed in 2 individuals after recruitment, but both were found to be positive for IgG against SARS‐CoV‐2.

^b^ Primary case: the individual who introduced the SARS‐CoV‐2 infection into the household. Secondary cases: household members infected by a primary case.

^c^ Viral strain was determined for 103 infected participants.

^d^ 1 uninfected participant not answering the question about underlying disease was categorized as having “no underlying disease”.

Viral strain was determined for 96.3% of cases; 63.1% were infected with non‐VOC (included May 2020–January 2021), and 36.9% with the Alpha B.1.1.7 variant (included March–April 2021) (Table 1). There were no differences in age, sex, or proportion with underlying disease between Alpha‐ and non‐VOC‐infected cases.

Viral load was 15.5‐fold higher in Alpha cases versus non‐VOC cases (*p* = 0.0001) (Table 1), while there was no difference between primary and secondary cases (Table 1). Onset of disease was earlier than the first study visit day (D0) for several cases (Table 1). For primary cases, the median time between the first positive PCR test and D0 was 3 days (*n* = 61). There was no difference between Alpha and non‐VOC cases in time since infection onset for any of the visit days (Tx days, Table 1 and data not shown).

### 4.2 Longitudinal Antibody Responses

**FIGURE 2:**
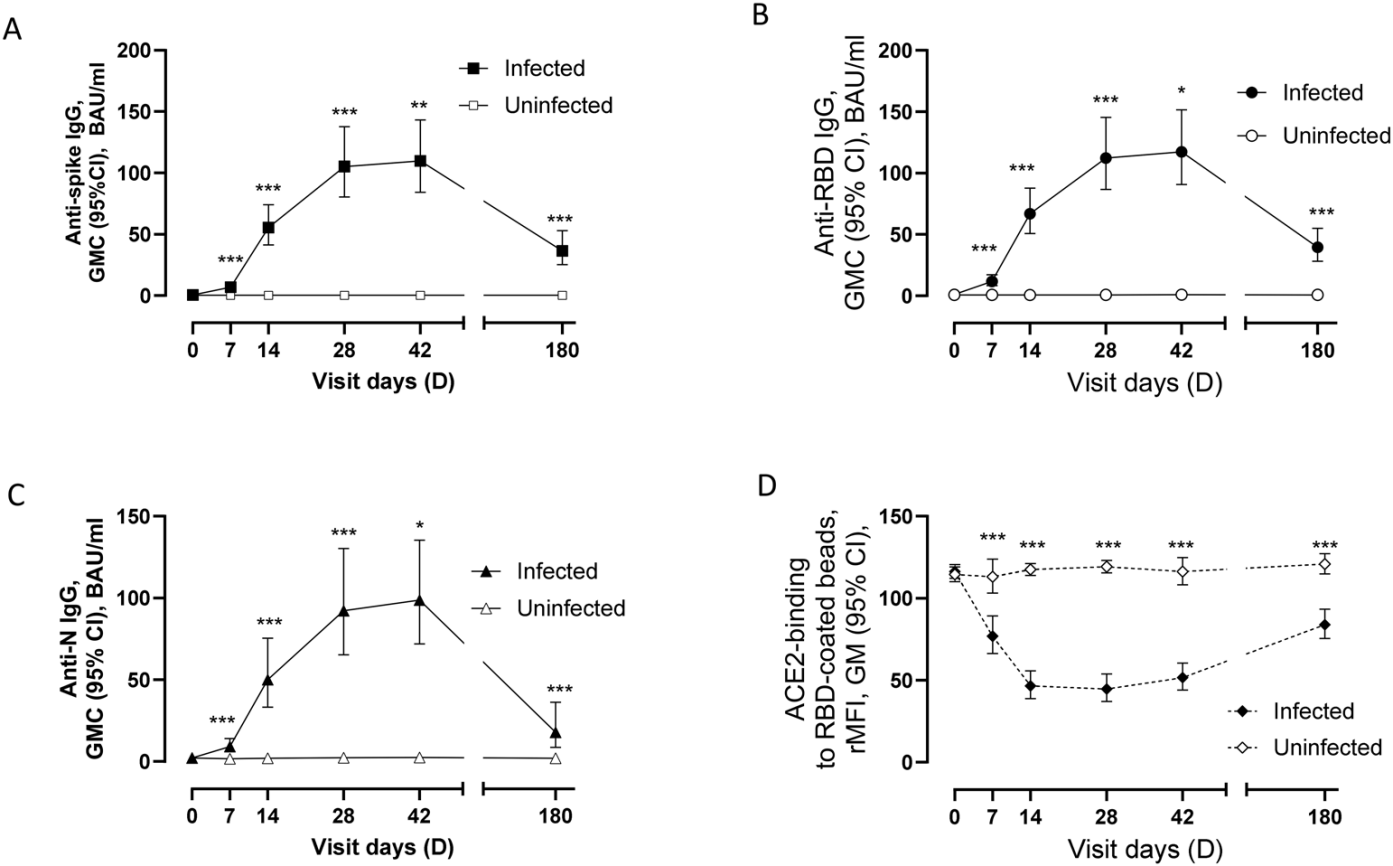
Longitudinal IgG responses in SARS‐CoV‐2 infected and uninfected study participants. (A) Binding antibodies against spike. (B) Binding antibodies against receptor binding domain (RBD). (C) Binding antibodies against nucleocapsid (N). (A–C) Significant differences between paired samples on consecutive visit days are shown. (D) Inhibition of ACE2‐RBD interaction. ACE2 binding to RBD‐coated beads after incubation with serum. Lower ACE2 binding indicates greater inhibition of ACE2‐RBD interaction. Significant differences between infected and uninfected samples are shown. BAU/mL, binding antibody units/mL; CI, confidence interval; GM, geometric mean; GMC, geometric mean concentration; rMFI, relative median fluorescent intensity. *p < 0.05, **p < 0.01, ***p ≤ 0.0003.

Overall, 690 sera were obtained during 6 study visits from inclusion until 6 months later (Table S1). IgG levels were significantly higher in infected compared to uninfected individuals at all time points after D0 (Figure 2). All cases developed antibodies against at least two of the SARS‐CoV‐2 antigens spike S1, RBD or N [24] between D0 and D42, and antibody levels against all three antigens increased significantly between consecutive visits for paired samples between D0 and D42, and declined between D42 and D180 (Figure 2A–C). Secondary cases showed a delay in antibody responses against all three antigens compared with primary cases, particularly on D7 (*p* ≤ 0.0001) (Figure S1). However, when analyzing antibody levels for these two groups according to median time since infection onset, the antibody trajectories appeared comparable (Figure S1).

From D7, sera from infected, but not from uninfected participants were shown to inhibit the ACE2‐RBD interaction (Figure 2D). Primary cases showed higher inhibition of RBD‐ACE2 interaction than secondary cases on D7 only (*p* < 0.0001) (data not shown).

### 4.3 Antibodies and Virus Variant

**FIGURE 3:**
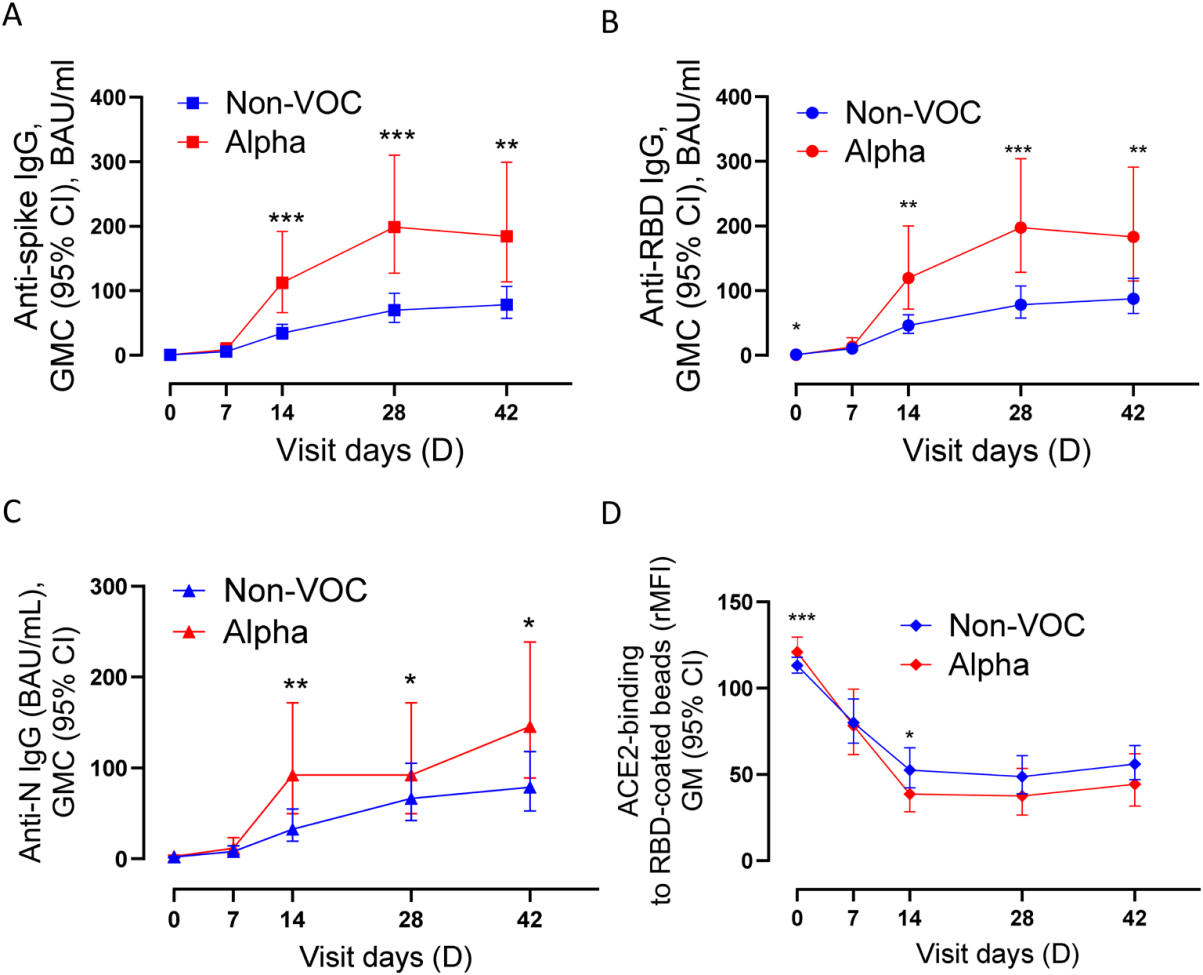
Longitudinal IgG responses after infection with Alpha B.1.1.7 or non‐VOC (non‐variant of concern). (A) Binding antibody levels against spike. (B) Binding antibody levels against receptor binding domain (RBD). (C) Binding antibody levels against nucleocapsid (N). (D) Inhibition of ACE2‐RBD interaction. ACE2‐binding to RBD‐coated beads after incubation with serum from non‐VOC or Alpha‐cases. Lower ACE2‐binding indicates greater inhibition of ACE2‐RBD interaction. BAU/mL, binding antibody units/mL; CI, confidence interval; GMC, geometric mean concentration; rMFI, relative median fluorescent intensity. *p < 0.05, **p < 0.001, ***p ≤ 0.0001.

Alpha‐cases developed significantly higher levels of binding antibodies compared to non‐VOC cases against S1, RBD and N between D14 and D42 (Figure 3A–C), with the largest difference on D14 (2.6–3.2‐fold higher for Alpha depending on antibody specificity). Alpha cases also displayed higher levels of antibodies inhibiting ACE2‐RBD interaction than non‐VOC cases, particularly on D14 (*p* = 0.046) (Figure 3D). Most Alpha cases had been vaccinated by D180; therefore D180 data were excluded.

**FIGURE 4:**
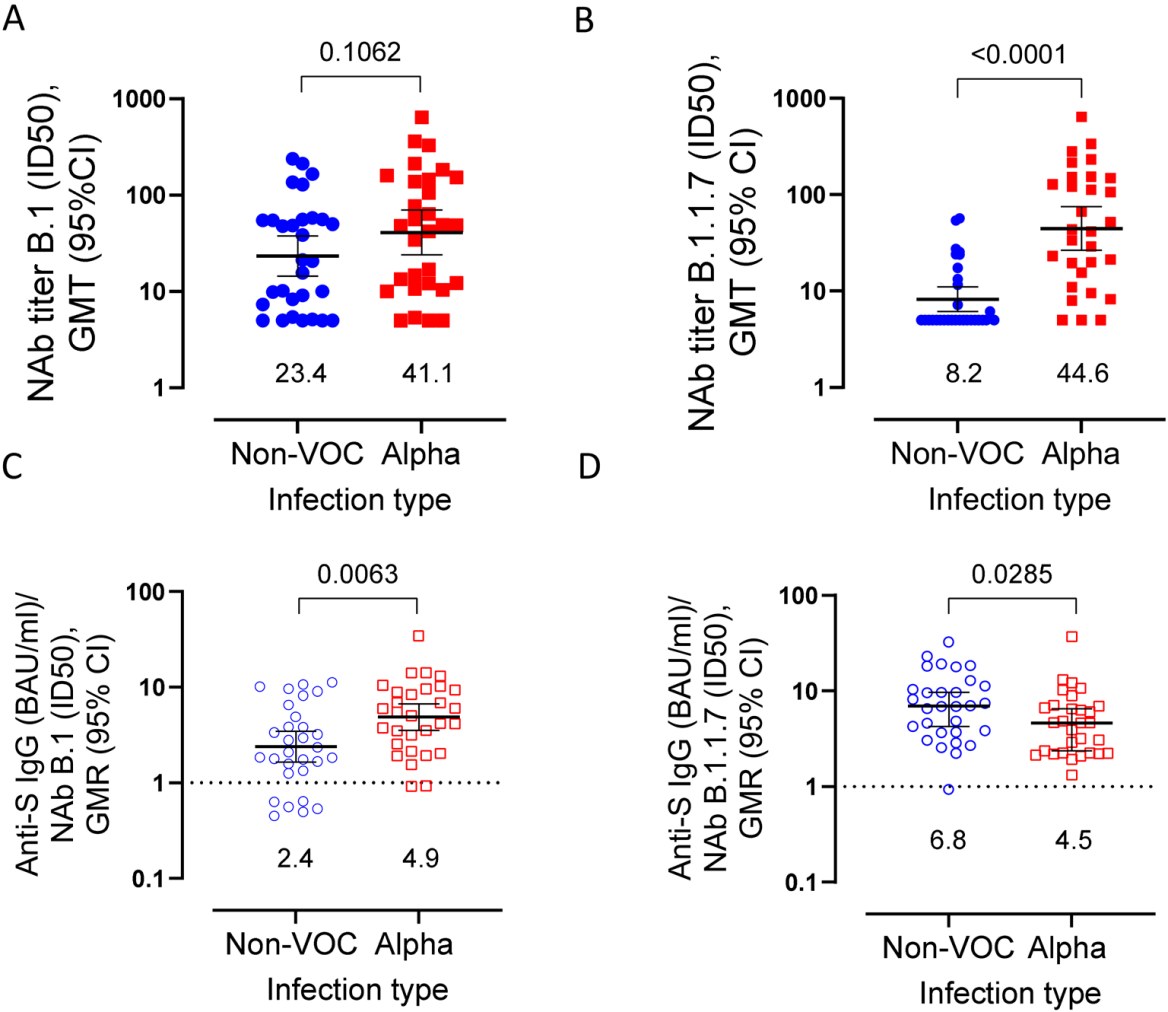
Functional antibody responses in D28 sera from SARS‐CoV‐2 cases infected with Alpha B.1.1.7 or non‐VOC (non‐variant of concern). (A) Neutralizing antibody (NAb) titer against Wuhan type B.1 virus. (B) NAb titer against Alpha virus B.1.1.7. (C) Ratio of spike (S)‐binding IgG to NAb titer against B.1 virus. (D) Ratio of S‐binding IgG to NAb titer against B.1.1.7. BAU/mL, binding antibody units/mL; CI, confidence interval; GMR, geometric mean ratio; GMT, geometric mean titer; ID50, median infective dose. The numbers below the dots indicate the geometric means of the measurement.

Two subsets of D28 sera were tested in neutralization assays against Wuhan B.1 and Alpha B.1.1.7 live viruses. D28 sera were chosen as this was the time point with the highest inhibition of ACE2‐RBD interaction among the infected cases (Figure 2C). Non‐VOC cases demonstrated a significant reduction in neutralizing antibody (NAb) titers against B.1.1.7, compared to B.1 (Figure S2A), whereas Alpha cases displayed similar NAb titers against both B.1 and B.1.1.7 (Figure S2B). Participants infected with Alpha displayed 1.8‐fold higher NAb titers against B.1 and 5.4‐fold higher NAb titers against B.1.1.7 than individuals infected with non‐VOC (Figure 4A,B, respectively).

To investigate the quality of the antibody response, we calculated the ratio of spike (S1)‐binding IgG level to the NAb titer at D28 whereby a higher ratio could imply induction of more non‐neutralizing antibodies [29]. Non‐VOC cases had significantly lower ratios than Alpha cases against the B.1. virus (Figure 4C), while the Alpha cases showed the lowest ratios against B.1.1.7. Almost identical results were obtained when calculating the ratio of anti‐RBD IgG levels to NAb (Figure S2C,D).

### 4.4 Antibodies and Viral Load

We investigated whether the maximum IgG levels (D28 or D42) against S1, RBD or N were associated with the viral load in the sample with the lowest Ct value. No association was found for any of the three antibody specificities (*n* = 97), nor if we restricted the analyses to secondary cases only (*n* = 39) (data not shown). Moreover, no correlation was found between viral load and NAb titers (*n* = 57, data not shown).

### 4.5 Antibodies in Relation to Self‐Reported Symptoms and Severity of Disease

For 11 out of 12 symptoms, infected participants reported significantly higher frequencies than uninfected participants (Table 2). The median number of symptoms among infected participants was 8; Alpha cases reported a median of 10 symptoms compared with 7 for non‐VOC cases (*p* = 0.013; Table 2). Alpha cases were more likely to report sore throat, loss of taste/smell, and stomach pain/diarrhea than non‐VOC cases (Table 2). A similar trend was observed for nausea. Primary and secondary cases reported comparable frequencies of symptoms (Table S2).

**TABLE 2:** Self‐reported symptoms according to infection status and virus variant.

	All study participants (*n* = 138)	Uninfected participants (*n* = 31)	Infected cases (*n* = 107)	*p*	Infected (*n* = 103^a^)		
					Non‐VOC (*n* = 65)	Alpha (*n* = 38)	*p*
Number of symptoms, median (range)	7 (0–12)	4 (0–7)	8 (1–12)	< **0.001**	7 (1–12)	10 (1–12)	**0.013**
Headache, *n* (%)	107 (77.5)	18 (58.1)	89 (83.2)	**0.003**	52 (80.0)	34 (89.5)	0.211
Stuffy or runny nose, *n* (%)	105 (76.1)	17 (54.8)	88 (82.2)	**0.002**	52 (80.0)	33 (86.8)	0.378
Cough, *n* (%)	96 (69.6)	8 (25.8)	88 (82.2)	< **0.001**	51 (78.5)	34 (89.5)	0.156
Tired, *n* (%)	92 (66.7)	12 (38.7)	80 (74.8)	< **0.001**	47 (72.3)	30 (79.0)	0.454
Muscle pain, *n* (%)	85 (61.6)	7 (22.6)	78 (72.9)	< **0.001**	44 (67.7)	32 (84.2)	0.066
Sore throat, *n* (%)	82 (59.4)	15 (48.4)	67 (62.6)	0.155	35 (53.9)	28 (73.7)	**0.046**
Chills, *n* (%)	80 (58.0)	9 (29.0)	71 (66.4)	< **0.001**	40 (61.5)	29 (76.3)	0.124
Loss of taste/smell, *n* (%)	71 (51.5)	2 (6.5)	69 (64.5)	< **0.001**	36 (55.4)	31 (81.6)	**0.007**
Fever, *n* (%)	55 (39.9)	3 (9.7)	52 (48.6)	< **0.001**	28 (43.1)	22 (57.9)	0.147
Trouble breathing, *n* (%)	54 (39.1)	3 (9.7)	51 (47.7)	< **0.001**	28 (43.1)	22 (57.9)	0.147
Stomach pain/diarrhea, *n* (%)	40 (29.0)	4 (12.9)	36 (33.6)	**0.025**	16 (24.6)	19 (50.0)	**0.009**
Nausea, *n* (%)	32 (23.2)	2 (6.5)	30 (28.0)	**0.012**	14 (21.5)	14 (36.8)	0.092

*Note:* *P*‐values < 0.05 in bold.

^a^ Virus variant was known for 103 of 107 cases.

**FIGURE 5:**
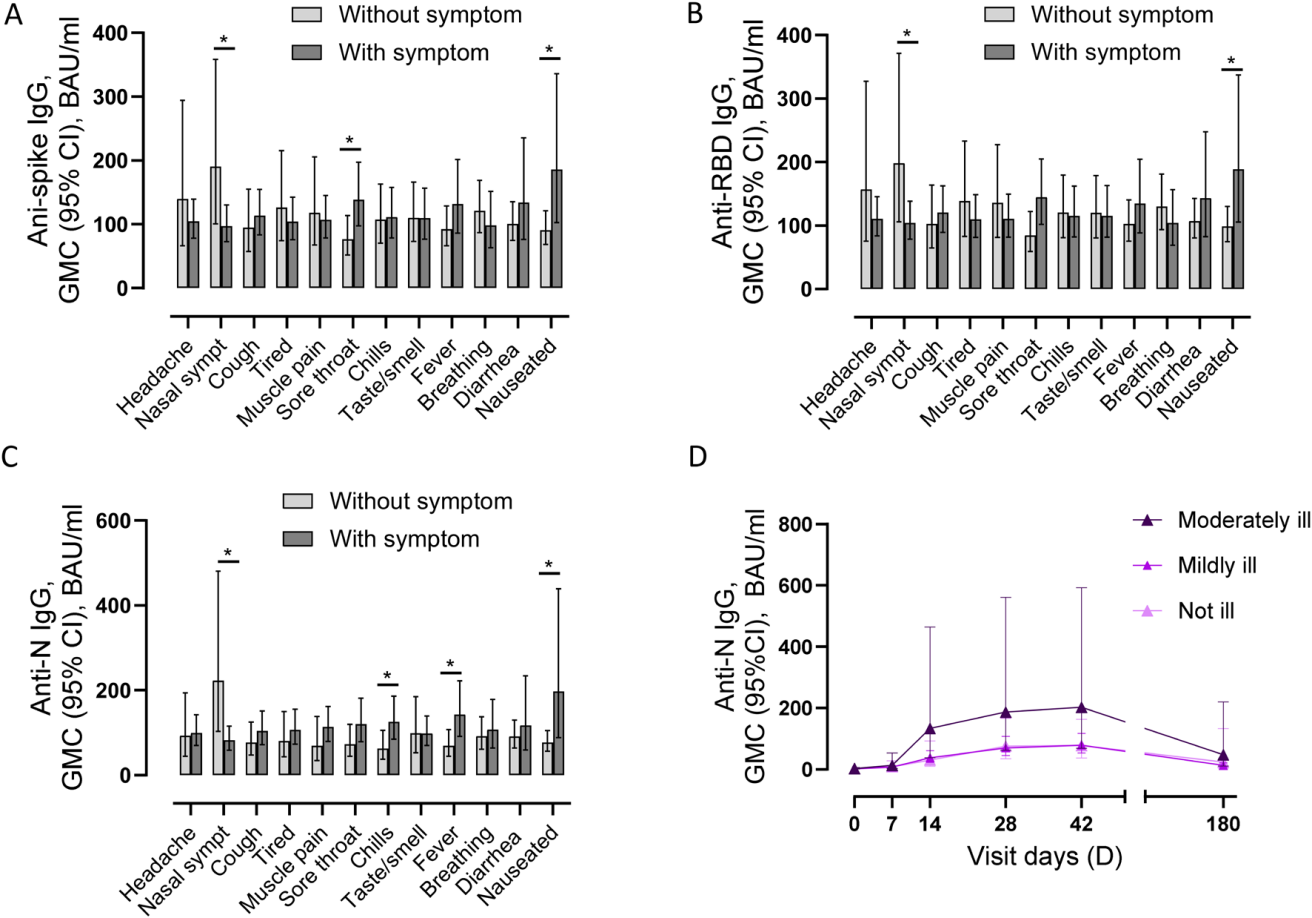
IgG levels according to self‐reported symptoms and severity of disease. For symptom analyses, D42 antibody levels are shown for infected cases with or without a specific symptom. (A) Binding antibodies against spike. (B) Binding antibodies against receptor binding domain (RBD) (C) Binding antibodies against nucleocapsid (N). (D) Antibodies levels against N according to severity category. Not ill: *N* = 6–12 cases/time point, mildly ill: *N* = 31–57 cases/time point, moderately ill: *N* = 10–21 cases/time point. BAU/mL, binding antibody units/mL; Breathing, trouble breathing; CI, confidence interval; GMC, geometric mean concentration; Taste/smell, loss of taste and/or smell. *p < 0.05.

We studied whether D42 antibody levels (plateau phase) were associated with reported symptoms. Higher anti‐spike and anti‐RBD IgG levels were observed in cases reporting nausea (28.0%) (*p* = 0.03 and 0.04, respectively) or a sore throat (62.2%) (*p* = 0.03 and 0.052, respectively) (Figure 5A,B; Figure S3A,B). Nausea was also associated with significantly higher anti‐N antibody levels (Figure 5C; Figure S3C). Individuals reporting fever (48.6%) or chills (66.4%) had higher levels of anti‐N IgG than individuals without these symptoms. Among individuals reporting fever, 86.5% also reported chills. Individuals with a runny/stuffy nose (82.2%) had lower levels of antibodies against all three antigens than cases without this symptom. We also grouped D42 IgG levels as low, intermediate and high (≤ 25 percentile, 25–75 percentile and ≥ 75 percentile, respectively) and studied symptom frequency according to these groups (Table S3). For all antibodies measured (anti‐S, anti‐RBD and anti‐N), symptoms were generally most frequent in the high antibody groups, although only significant for cough (all antibody types), nausea (anti‐spike and anti‐N IgG) and fever (anti‐RBD and anti‐N IgG). Symptoms from the nose were more common among individuals with low levels of anti‐N antibodies.

With respect to disease severity, most cases were scored as mildly ill (63.7%), whereas 23.1% were moderately ill and 13.2% were not ill. Neither virus type nor viral load was associated with disease severity (Table S4). However, the median number of symptoms was 5.5 in the not ill group and 10 in the moderately ill group (*p* = 0.0001; Table S4), and 6 of 12 symptoms were more frequently reported with more severe disease (Table S5). Time since infection onset did not differ by severity group for any visit day (data not shown).

No significant differences in IgG levels were found between the three severity groups, but the anti‐N antibody trajectory for the moderately ill group seemed higher compared to the other groups (Figure 5D and data not shown). There was no significant difference in levels of antibodies inhibiting the ACE‐RBD interaction (data not shown). The number of sera per severity group and visit days are shown in Table S6.

### 4.6 Predictors of Antibody Levels

**FIGURE 6:**
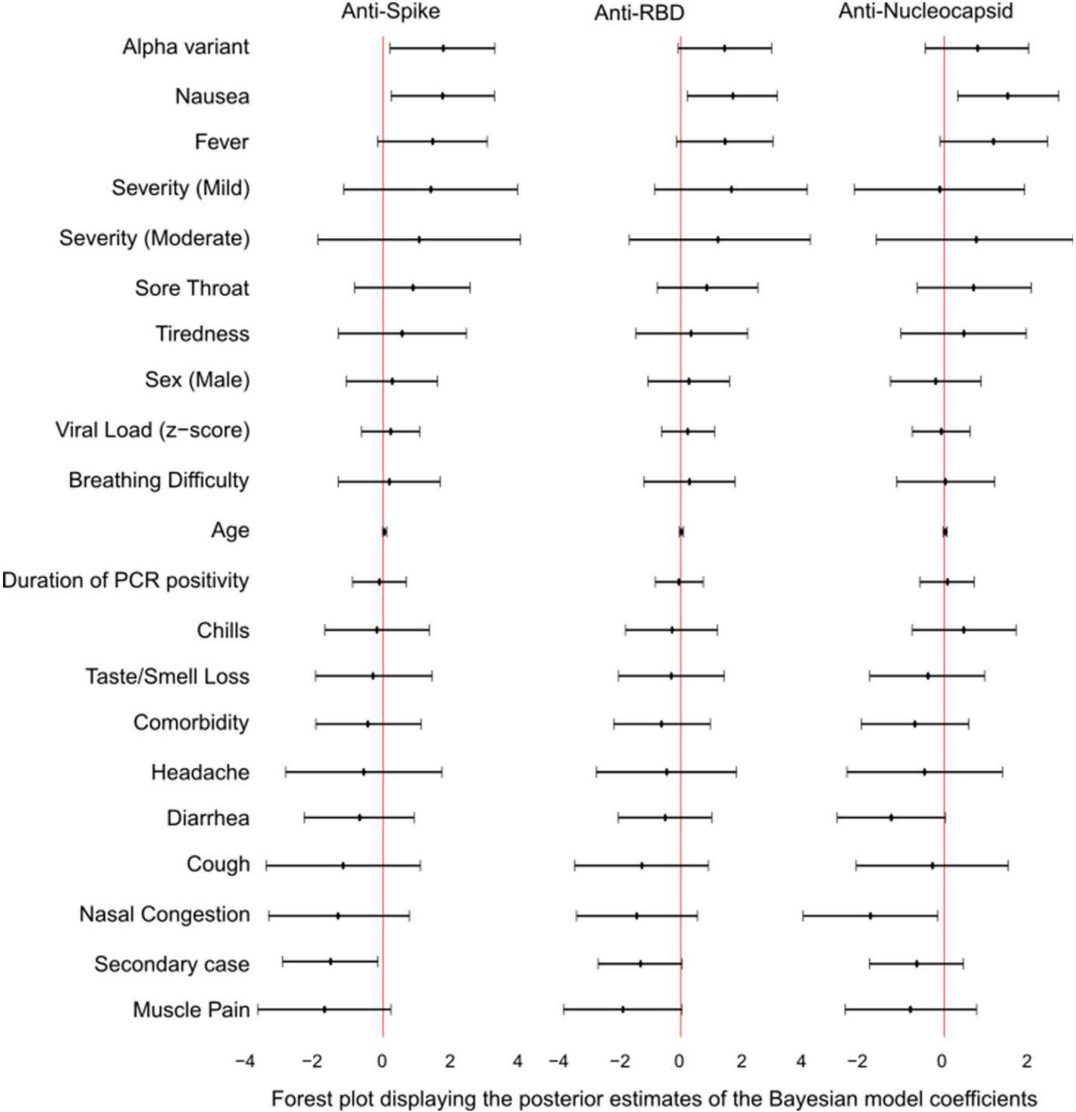
Forest plots showing associations between IgG level changes (from Day 0 to 42) and various influencing factors, as estimated using longitudinal Bayesian multilevel models. Point estimates with 95% credible intervals (CrI) are shown. RBD, Receptor binding domain. For symptoms, the group without the specific symptom is the reference. For severity, the group defined as “not ill” is the reference.

IgG levels against the three viral antigens demonstrated significant temporal variability and inter‐individual variability in Bayesian multilevel models (Figure 6; Table S7).

There was a significant positive association between anti‐spike IgG levels and the Alpha variant (1.80, 95% CrI: 0.20–3.35) and with nausea (1.78, 95% CrI: 0.24–3.33), and significant negative associations with being a secondary case (−1.58, 95% CrI: −3.03 to −0.16) (Figure 6; Table S7). Fever showed non‐significant positive effects on antibody levels (1.48, 95% CrI: −0.17 to 3.12), whereas muscle pain showed a negative trend (−1.77, 95% CrI: −3.78 to 0.22). Similar findings were found for RBD IgG levels (Figure 6; Table S7).

The Alpha variant showed a non‐significant positive association with anti‐N IgG levels (0.82, 95% CrI: −0.45 to 2.07) (Figure 6; Table S7). Nausea had a significant positive association (1.55, 95% CrI: 0.33–2.80) and fever a borderline positive association (1.21, 95% CrI: −0.10 to 2.52), while nasal symptoms and diarrhea displayed negative associations ((−1.79, 95% CrI: −3.43 to −0.15) and (−1.28, 95% CrI: −2.60 to 0.03), respectively).

Other factors were not associated with IgG levels (Figure 6; Table S7). The residual variability sigma indicates moderate to relatively low unexplained variance, and excellent diagnostics (Rhat≈1.00 for all antigens, high ESS) indicate robust parameter estimation (Table S7).

## 5 Discussion

Here we have compared longitudinal antibody responses induced by Alpha and non‐VOC infection in unvaccinated and previously unexposed individuals. To our knowledge, no other studies have explored this aspect, probably because Alpha virus circulation and the COVID‐19 vaccination campaign coincided worldwide, limiting the time window to study differences between non‐VOC and Alpha infections in naïve individuals, as the focus rapidly changed to subsequent VOCs and vaccinated study populations.

We found that all PCR‐confirmed cases were positive for IgG antibodies against SARS‐CoV‐2, and that IgG levels were higher in Alpha compared to non‐VOC cases both in univariate and multilevel analyses. A similar pattern was observed for antibodies inhibiting ACE2‐RBD interaction as well as for NAb titers against both B.1 and B.1.1.7. While disease severity was not associated with Alpha infection, Alpha cases reported a higher frequency of several symptoms compared to non‐VOC cases, e.g., loss of taste and smell confirming our previous findings [6].

Sera from non‐VOC cases had significantly lower NAb titers against the B.1.1.7 than B.1, indicating immune evasion by the mutated Alpha virus, while sera from Alpha cases neutralized both virus types equally well. In contrast, a study on convalescent sera from spring 2020, found similar neutralizing activity against wild type virus and B.1.1.7 [30]. Although Alpha cases had higher NAb titers against both viruses, the ratio of anti‐spike binding to NAb titer was lower against the infecting virus type for both non‐VOC and Alpha cases, which could imply fewer non‐neutralizing antibodies against the infecting virus type [29]. Furthermore, this may suggest that the increase of NAb observed in the Alpha cases was due to higher levels of antibodies and not necessarily antibodies with better neutralizing properties. However, as we only measured IgG antibodies, and no other isotypes, it could also indicate differences in immunoglobulin class distribution induced by the two viral variants.

Although Alpha‐cases had both higher IgG levels and higher viral loads, we found no association (univariate or multilevel) between these two variables for any of the three SARS‐CoV‐2 antibody specificities. Some studies have found that antibody levels against SARS‐CoV‐2 are associated with viral load [12, 13], whereas others have not [31]. Since SARS‐CoV‐2 viral load usually peaks before or during symptom onset [32], we probably missed the peak in several participants, and a potential association between serum IgG and viral load cannot therefore be ruled out. Moreover, we did not find an association between viral load and disease severity, but existing literature is also conflicting regarding this issue [13, 33].

Symptoms from the gastrointestinal tract and nausea are known to be associated with SARS‐CoV‐2 infection [34]. Here, Alpha‐cases reported higher frequencies of diarrhea/stomach pain and nausea than non‐VOC cases. Nausea was associated with higher IgG levels both in the univariate analyses with D42 sera, and in the longitudinal multilevel analyses. Amjadi et al. reported a similar frequency of nausea (34%) among COVID‐19 convalescent cases and found that nausea was correlated with higher antibody levels [22]. SARS‐CoV‐2‐specific antibody levels have been shown to be associated with fever [19, 22, 35]. We found that fever and chills were associated with higher anti‐N IgG levels 6 weeks post‐infection, but a trend was found for fever only (all antibody specificities) in the longitudinal multilevel analyses.

Other studies have shown a positive association between anti‐N antibodies and clinical severity of disease, like hospitalization [19, 21, 22]. In our study there was a trend that the most severe group had higher levels of anti‐N antibodies, but the confidence intervals were wide making it difficult to conclude, also in the multilevel analyses. We did not find an association between severity and virus variant, despite the observation that Alpha cases reported a higher number of symptoms than non‐VOC cases. In contrast, studies from the UK have shown higher severity and mortality due to Alpha infection than infection with earlier variants [10, 11]. However, our sample size was small when stratified based on virus type and all cases had relatively mild disease not requiring hospitalization.

The strength of this prospective study is the comprehensive longitudinal serum and virus sampling from individuals with PCR‐confirmed and virus‐typed SARS‐CoV‐2 infection, as well as sampling of uninfected participants. Moreover, the timing of the study allowed us to compare infection with different virus variants. All participants were immunologically naïve for SARS‐CoV‐2 prior to infection, meaning that the background immunity for all participants was comparable. Close monitoring of symptoms in all participants through self‐reported questionnaires enabled linkage to the biological data. Furthermore, the household study design enabled the investigation into relatively mild disease in non‐hospitalized cases, as opposed to many other studies based on hospital admissions.

A limitation of the study is the quite limited age range of the participants with few elderly cases and mostly adult primary cases. There was some loss to follow up at D180, and due to the introduction of the COVID‐19 vaccines, many samples from Alpha‐cases had to be excluded at D180. All symptoms and clinical conditions were self‐reported and prone to bias. However, since both the median number of symptoms as well as frequencies of many specific symptoms increased with increasing severity category, there was consistency in the reporting.

The multiplex method measuring IgG levels was based on ancestral Wuhan‐type antigens. Moreover, the assay standard was made of pooled plasma samples from recovered COVID‐19 cases between April and May 2020, meaning that no Alpha‐specific antibodies were present. Therefore, one could expect that the assays would be better at detecting antibodies in individuals infected with non‐VOC than Alpha, which is contrary to our observation. We also obtained similar differences between non‐VOC and Alpha sera using a previously published method during assay validation [25], as well as in the ACE2‐RBD interaction inhibition assay. Both of the latter assays were performed by flow cytometry using beads with Wuhan‐type antigens. The results likely reflect the close similarity of the Wuhan and Alpha variants [4], and that the majority of spike‐specific IgG antibodies bind to epitopes that are conserved between the two strains. Moreover, Alpha cases displayed higher neutralizing titers against Wuhan type B.1 virus than non‐VOC cases, corresponding well with the binding assays.

While the Alpha virus was the first VOC, the emergence of the Omicron variant in late 2021 was associated with increased infectivity and caused the majority of infections in Norway during the pandemic [36]. Infections with the Omicron variant were however associated with decreased disease severity [37], in contrast to the Alpha variant which has been suggested to be associated with more severe infection [38]. The present study shows that both the viral loads and the immune responses were higher among Alpha‐infected than non‐VOC‐infected cases. The induction of more powerful immune responses may have contributed to the increase in hospitalization and deaths observed after the emergence of the Alpha variant [8, 9, 11]. Although the Alpha variant is no longer in circulation, our study adds important information about an early stage in SARS‐CoV‐2 development and details how SARS‐CoV‐variants can differ in their pathogenesis and subsequent immune responses. With the continued evolution of novel variants, most recently, descendants of BA.2.86, it will be important to monitor potential changes in disease severity associated with these emerging variants. However, the observations presented here are based on a naïve population early in the pandemic. The far more complex immunity patterns currently seen towards SARS‐CoV‐2 obtained from both repeated infections and vaccination make it more difficult to elucidate factors causing differences in antibody responses to specific variants.

## 6 Conclusions

This study of unvaccinated individuals with relatively mild primary PCR‐confirmed COVID‐19 conducted in Norwegian households shows higher antibody responses induced by the more transmissible Alpha variant compared to earlier non‐VOC viruses. Nausea was also associated with higher antibody levels. Alpha cases were more likely to report sore throat, loss of taste/smell and stomach pain/diarrhea than non‐VOC cases.

## Funding

This research received no external funding and was solely funded by the Norwegian Institute of Public Health (NIPH).

## Ethics Statement

The study was approved by the Regional Ethics Committee in Norway (#118354).

## Consent

Written informed consent was obtained from the participants before study inclusion.

## Conflicts of Interest

The authors declare no conflicts of interest.

## Supporting information


**Figure S1:** Longitudinal antibody responses in SARS‐CoV‐2 infected primary and secondary cases. (A) Binding antibodies against spike according to visit days. (B) Binding antibodies against spike according to the median time since infection onset for the exposure group. (C) Binding antibodies against receptor binding domain (RBD) according to visit days. (D) Binding antibodies against RBD according to the median time since infection onset for the exposure group. (E) Binding antibodies against nucleocapsid (N) according to visit days. (D) Binding antibodies against N according to the median time since infection onset for the exposure group. BAU/mL, binding antibody units/mL; CI, confidence interval; GMC, geometric mean concentration. *p < 0.05, **p < 0.01, ***p ≤ 0.0003. Primary case: the individual who introduced the SARS‐CoV‐2 infection into the household. Secondary cases: household members infected by a primary case.
**Figure S2:** Functional Ab responses in D28 serum from SARS‐CoV‐2 cases infected with Alpha B.1.1.7 or non‐VOC (non‐variant of concern). (A) Neutralizing antibody (NAb) titer against Wuhan type B.1 and Alpha B.1.1.7 viruses among non‐VOC‐cases. (B) Nab titer against Wuhan type B.1 and Alpha B.1.1.7 viruses among Alpha‐cases. Paired samples are connected by lines. (C) Ratio of RBD‐binding IgG to NAb titer against B.1 virus. (D) Ratio of RBD‐binding IgG to NAb titer against B.1.1.7. BAU/mL, binding antibody units/mL; CI, confidence interval; GMR, geometric mean ratio; GMT, geometric mean titer; ID50, median infective dose; RBD, receptor binding domain. The numbers above the dots indicate the geometric mean of the measurement.
**Figure S3:** IgG levels according to self‐reported symptoms. D42 antibody levels are shown as box plots with whiskers from minimum to maximum for infected cases with or without a specific symptom. Dots represent individual values. (A) Binding antibodies against spike. (B) Binding antibodies against receptor binding domain (RBD). (C) Binding antibodies against nucleocapsid (N). BAU/mL, binding antibody units/mL; Breathing, trouble breathing; Taste/smell, loss of taste and/or smell. *p < 0.05.

**Data S1:** apm70102‐sup‐0002‐DataS1.docx.

**Table S1:** Overview of samples tested for antibodies binding to SARS‐CoV‐2 for different exposure groups.
**Table S2:** Frequencies of self‐reported symptoms for primary and secondary cases and females and males.
**Table S3:** Frequency of symptoms among infected individuals with various antibody levels on visit day 42.
**Table S4:** Characteristics of the severity groups.
**Table S5:** Frequencies of self‐reported symptoms for the various severity groups.
**Table S6:** Overview of samples tested for antibodies binding to SARS‐CoV‐2 for the severity groups.

**Table S7:** Regression coefficients and smoothing spline hyperparameters from Bayesian regression models for gaussian family.

## Data Availability

The data presented in this study are available on request from the corresponding author. The data are not publicly available due to regulations in the Norwegian Health Research Act and the Norwegian Data Protection Act for use (and storage) of Personal Data related to health. Access to data can only be given to applicants with an ethical approval from their IRB or equivalent body, and an exemption from the duty of confidentiality from Health Registry controllers in Norway.
