# Supplementary material for: SARS‐CoV‐2 Infection With Alpha B.1.1.7 Virus Induced Higher Antibody Responses Than Earlier Non‐VOC Variants During the First Waves of the COVID‐19 Pandemic in Norway
Source: APMIS. 2025 Dec 10;133(12):e70102. doi: 10.1111/apm.70102 (PMC12696439; doi:10.1111/apm.70102)
Supplement: Supplementary file 2 — Data S1: apm70102‐sup‐0002‐DataS1.docx. [file APM-133-0-s003.docx]

**Supplementary methods**

To investigate factors influencing IgG levels collected longitudinally from infected participants between D0 and D42, we utilized brms package version 2.22.0 in R which fits complex Bayesian multilevel models using Stan^1^ (one model per antibody specificity). BoxCox transformed IgG level was specified as the outcome and included standardized age and viral load as continuous predictors, smooth terms for time-varying effects, and virus variant, sex, being a primary or secondary case, severity, comorbidity, fever, cough, and other symptoms as categorical predictors, and random effects to account for within-individual correlations.

The primary model was specified as follows:

$y_{i}j=\beta_{0}+\Sigma\left( \beta_{k}*X_{k}ij \right)+\Sigma\left( f_{m}\left( Z_{m}ij \right) \right)+\Sigma\left( \gamma_{n}*F_{n}ij \right)+u_{j}+\varepsilon_{i}j$

Where:

$y_{ij}$ is BoxCox transformed measure of serum antibody level stratified by antigen

$\beta_{0}$ is the intercept

$\beta_{k}$ are coefficients for continuous predictors $X_{kij}$

captures the non-linear effects of time on an$\Sigma\left( f_{m}\left( Z_{m}ij \right) \right)$

$\gamma_{n}$ are coefficients for categorical predictors $F_{n}ij$

$u_{j}$ is random effect for participant $j$

$\epsilon_{ij}$ is residual error term

The smooth functions were modeled using thin plate regression splines with the default parameters in the mgcv package version 1.9-1^2^. We employed weakly informative normal priors for all parameters with mean 0 and standard deviation 10 for predictors and Half-Cauchy priors with location 0 and scale 5 for random effects and smoothing parameters. Model fit was evaluated using posterior predictive checks and posterior means and 95% credible intervals (CrI) for all parameters are reported.

**References**

1. Carpenter B, Gelman A, Hoffman MD, et al. Stan: A Probabilistic Programming Language. *Journal of Statistical Software.* 2017;76(1):1 - 32.

2. Wood SN. *Generalized Additive Models: An Introduction with R, Second Edition.* New York: Chapman and Hall/CRC; 2017.
