# Supplementary material for: SARS‐CoV‐2 Infection With Alpha B.1.1.7 Virus Induced Higher Antibody Responses Than Earlier Non‐VOC Variants During the First Waves of the COVID‐19 Pandemic in Norway
Source: APMIS. 2025 Dec 10;133(12):e70102. doi: 10.1111/apm.70102 (PMC12696439; doi:10.1111/apm.70102)
Supplement: Supplementary file 3 — Table S1: Overview of samples tested for antibodies binding to SARS‐CoV‐2 for different exposure groups. Table S2: Frequencies of self‐reported symptoms for primary and secondary cases and females and males. Table S3: Frequency of symptoms among infected individuals with various antibody levels on visit day 42. Table S4: Characteristics of the severity groups. Table S5: Frequencies of self‐reported symptoms for the various severity groups. Table S6: Overview of samples tested for antibodies binding to SARS‐CoV‐2 for the severity groups. [file APM-133-0-s002.docx]

**Supplementary Table 1. Overview of samples tested for antibodies binding to SARS-CoV-2 for different exposure groups**

|  | **All (n=138)** | **Uninfected (n=31)** | **Infected (n=107)** | **Infected cases** | | | |
| --- | --- | --- | --- | --- | --- | --- | --- |
|  |  |  |  | **Primary cases† (n=64/33††)** | **Secondary cases† (n=43/19††)** | **Non-VOC**  **infected (n=65/49††)** | **Alpha infected**  **(n=38/1††)** |
| **D0, n (%)** | 131 (94.9) | 30 (96.8) | 101 (94.4) | 60 (93.8) | 41 (95.3) | 61 (93.8) | 37 (97.4) |
| **D7, n (%)** | 128 (92.8) | 25 (80.6) | 103 (96.3) | 63 (98.4) | 40 (93.0) | 62 (95.4) | 37 (97.4) |
| **D14, n (%)** | 125 (90.6) | 24 (77.4) | 101 (94.4) | 61 (95.3) | 40 (93.0) | 61 (93.8) | 36 (94.7) |
| **D28, n (%)** | 119 (86.2) | 22 (71.0) | 97 (90.7) | 58 (90.6) | 39 (90.7) | 59 (90.8) | 35 (92.1) |
| **D42, n (%)** | 119 (86.2) | 25 (80.6) | 94 (87.9) | 57 (89.1) | 37 (86.0) | 57 (87.7) | 33 (86.8) |
| **D180†, n (%)** | 68 (49.3) | 16 (51.6) | 52 (48.6) | 33 (100) | 19 (100) | 49 (100) | 1  (100) |

**†**Primary case: the individual who introduced the SARS-CoV-2 infection into the household. Secondary cases: household members infected by a primary case.

†**†**Only samples from unvaccinated individuals were included.

**Supplementary Table 2. Frequencies of self-reported symptoms for primary and secondary cases and females and males**

|  | **Infected cases (n=107)** | | | | | |
| --- | --- | --- | --- | --- | --- | --- |
|  | **Primary case† (n=64)** | **Secondary case† (n=43)** | **p-value** | **Female**  **(n=54)** | **Male**  **(n=53)** | **P-value** |
| **Number of symptoms, median (range)** | 8 (1-12) | 7 (1-12) | 0.647 | 8 (1-12) | 8 (1-12) | 0.460 |
| **Cough, n (%)** | 51 (79.7) | 37 (86.1) | 0.399 | 43 (79.6) | 45 (84.9) | 0.475 |
| **Muscle pain, n (%)** | 48 (75.0) | 30 (69.8) | 0.550 | 41 (75.9) | 37 (69.8) | 0.477 |
| **Chills, n (%)** | 45 (70.3) | 26 (60.5) | 0.291 | 32 (59.3) | 39 (75.6) | 0.117 |
| **Loss of taste and/or smell, n (%)** | 44 (68.8) | 25 (58.1) | 0.261 | 34 (63.0) | 35 (66.0) | 0.740 |
| **Fever, n (%)** | 35 (54.7) | 17 (39.5) | 0.124 | 25 (48.2) | 26 (49.1) | 0.925 |
| **Trouble breathing, n (%)** | 32 (50.0) | 19 (44.2) | 0.555 | 26 (48.2) | 26 (49.1) | 0.925 |
| **Headache, n (%)** | 53 (82.8) | 36 (83.7) | 0.902 | 46 (85.2) | 43 (81.1) | 0.575 |
| **Stuffy or runny nose, n (%)** | 52 (81.3) | 36 (83.7) | 0.743 | 46 (85.2) | 42 (79.3) | 0.421 |
| **Tired, n (%)** | 49 (76.6) | 31 (72.1) | 0.602 | 42 (77.8) | 38 (71.7) | 0.469 |
| **Nauseated/**  **vomiting, n (%)** | 19 (29.7) | 11 (25.6) | 0.643 | 17 (31.5) | 13 (24.5) | 0.423 |
| **Stomach pain/ Diarrhoea, n (%)** | 22 (34.4) | 14 (32.6) | 0.845 | 21 (38.9) | 15 (28.3) | 0.247 |
| **Sore throat, n (%)** | 39 (60.9) | 28 (65.1) | 0.661 | 34 (63.0) | 33 (62.3) | 0.940 |

**†**Primary case: the individual who introduced the SARS-CoV-2 infection into the household. Secondary cases: household members infected by a primary case.

**Supplementary Table 3. Frequency of symptoms among infected individuals with various antibody levels on visit day 42**

|  | IgG levels**†** | | | | | | | | | | | | |
| --- | --- | --- | --- | --- | --- | --- | --- | --- | --- | --- | --- | --- | --- |
|  | Anti-spike | | | | Anti-RBD | | | | Anti-Nucleocapsid | | | | |
|  | Low | Intermediate | High | p-value‡ | Low | Intermediate | High | p-value‡ | Low | Intermediate | High | p-value‡ | |
| Headache (%), n¶ | 83.3, 20 | 84.8, 39 | 84.6, 11 | 0.84 | 83.3, 20 | 84.8, 39 | 79.2, 19 | 0.84 | 91.7, 22 | 80.4, 37 | 79.2, 19 | | 0.42 |
| Nasal symptoms (%), n | 83.3, 20 | 84.8, 39 | 75.0, 18 | 0.59 | 83.3, 20 | 84.8, 39 | 75.0, 18 | 0.59 | 100.0, 24 | 78.3, 36 | 70.8, 17 | | **0.021** |
| Cough (%), n | 87.5, 21 | 71.7, 33 | 95.8, 23 | **0.032** | 87.5, 21 | 71.7, 33 | 95.8, 23 | **0.032** | 91.7, 22 | 71.7, 33 | 91.7, 22 | | **0.043** |
| Tired (%), n | 75.0, 18 | 69.6, 32 | 75.0, 18 | 0.84 | 75.0, 18 | 69.6, 32 | 75.0, 18 | 0.84 | 70.8, 17 | 69.6, 32 | 79.2, 19 | | 0.68 |
| Muscle pain (%), n | 75.0, 18 | 73.9, 34 | 62.5, 15 | 0.54 | 79.2, 19 | 69.6, 32 | 84.6, 16 | 0.59 | 54.2, 13 | 78.3, 36 | 75.0, 18 | | 0.096 |
| Sore throat (%), n | 50.0, 12 | 56.5, 26 | 76.9, 10 | 0.086 | 58.3, 14 | 52.2, 24 | 79.2, 19 | 0.087 | 58.3, 14 | 52.2, 24 | 79.2, 19 | | 0.087 |
| Chills (%), n | 70.8, 17 | 56.5, 26 | 75.0, 18 | 0.24 | 79.2, 19 | 52.2, 24 | 75.0, 18 | **0.039** | 50.0, 12 | 63.0, 29 | 83.3, 20 | | 0.050 |
| Taste/smell (%), n | 62.5, 15 | 58.7, 27 | 62.5, 15 | 0.93 | 66.7, 16 | 56.5, 26 | 62.5, 15 | 0.70 | 58.3, 14 | 60.9, 15 | 62.5, 15 | | 0.96 |
| Fever (%), n | 54.2, 13 | 39.1, 18 | 62.5, 15 | 0.15 | 58.3, 14 | 34.8, 16 | 66.7, 16 | **0.023** | 29.2, 7 | 50.0, 23 | 66.7, 16 | | **0.033** |
| Breathing (%), n | 62.5, 15 | 39.1, 18 | 45.8, 11 | 0.18 | 58.3, 14 | 41.3, 19 | 45.8, 11 | 0.40 | 50.0, 12 | 43.5, 20 | 50.0, 12 | | 0.82 |
|  |  |  |  |  |  |  |  |  |  |  |  | |  |
| Diarrhea (%), n | 29.2, 7 | 30.4, 14 | 33.3, 8 | 0.95 | 25.0, 6 | 30.4, 14 | 37.5, 9 | 0.642 | 33.3, 8 | 23.9, 11 | 41.7, 10 | | 0.30 |
| Nausea (%), n | 8.7, 2 | 28.3, 13 | 41.7, 10 | **0.031** | 12.5, 3 | 26.1, 12 | 41.7, 10 | **0.073** | 20.8, 5 | 19.6, 9 | 45.8, 11 | | **0.047** |

**†**IgG levels are given as Low: ≤25 percentile, Intermediate: 25-75 percentile, high: ≥75 percentile

‡Chi-squared test, bold text, p<0.05

¶Individuals with symptom

**Supplementary Table 4. Characteristics of the severity groups**

|  | **Infected with severity score (n=91)** | | | | |
| --- | --- | --- | --- | --- | --- |
|  | **Not ill (n=12, 13.2%)** | **Mildly ill (n=58, 63.7%)** | **Moderately ill (n=21, 23.1%)** | **p-value** |  |
| Age (years), median (IQR†) | 36.5 (26.5-47.5) | 40.5 (32-48) | 35 (31-45) | 0.380 |  |
| Females, n (%) | 6 (50) | 26 (44.8) | 14 (66.7) | 0.230 |  |
| Underlying disease, n (%) | 3 (25) | 15 (25.9) | 5 (23.8) | 0.983 |  |
| Overweight (BMI ≥25), n (%) | 0 | 4 (6.9) | 1 (4.8) | 0.625 |  |
| Primary case, n (%) | 5 (41.7) | 19 (32.8) | 10 (47.6) | 0.457 |  |
| Infected with non-VOC‡, n (%) | 8 (14.0) | 37 (64.9) | 12 (21.1) | 0.767 | |
| Infected with Alpha‡, n (%) | 3 (10.0) | 19 (63.3) | 8 (26.7) |  |  |
| Viral load (RNA copies/µL eluate), GMC (95%CI)(n)**¶** | 616 (26-14,696(8) | 955 (398-2,289) (55) | 808 (240-2,726) (21) | 0.457 |  |
| Number of symptoms, median (range) | 5.5 (1-9) | 8 (1-12) | 10 (5-12) | **0.0001** |  |

†Interquartile range

‡Viral strain was missing for 1 not ill case, 2 mildly ill cases and 1 moderately ill case.

¶GMC: Geometric mean concentration, CI: Confidence interval

**Supplementary Table 5. Frequencies of self-reported symptoms for the various severity groups**

|  | **Infected with severity score (n=91)** | | | |
| --- | --- | --- | --- | --- |
|  | **Not ill, n=12 (13.2%)** | **Mildly ill, n=58 (63.7%)** | **Moderately ill, n=21 (23.1%)** | **P-value** |
| **Number of symptoms, median (range)** | 5.5 (1-9) | 8 (1-12) | 10 (5-12) | **0.0001** |
| **Cough, n (%)** | 6 (50.0) | 52 (89.7) | 21 (100.0) | **<0.001** |
| **Muscle pain, n (%)** | 4 (33.3) | 46 (79.3) | 18 (85.7) | **0.002** |
| **Chills, n (%)** | 5 (41.7) | 40 (69.0) | 17 (81.0) | 0.065 |
| **Loss of taste and/or smell, n (%)** | 6 (50.0) | 37 (63.8) | 16 (76.2) | 0.305 |
| **Trouble breathing, n (%)** | 3 (24.0) | 31 (53.5) | 12 (57.1) | 0.158 |
| **Fever, n (%)** | 3 (25.0) | 31 (53.5) | 12 (57.1) | 0.158 |
| **Headache, n (%)** | 9 (75.0) | 50 (86.2) | 21 (100.0) | 0.085 |
| **Stuffy or runny nose, n (%)** | 11 (91.7) | 48 (82.8) | 19 (90.5) | 0.563 |
| **Tired, n (%)** | 6 (50.0) | 42 (72.4) | 21 (100.0) | **0.003** |
| **Nausea/vomited, n (%)** | 2 (16.7) | 14 (24.1) | 11 (52.4) | **0.030** |
| **Stomach pain/ Diarrhoea. n (%)** | 1 (8.3) | 20 (34.5) | 12 (57.1) | **0.018** |
| **Sore throat, n (%)** | 4 (33.3) | 36 (62.1) | 17 (81.0) | **0.024** |

**Supplementary Table 6. Overview of samples tested for antibodies binding to SARS-CoV-2 for the severity groups**

|  |  | **Severity score** | | |
| --- | --- | --- | --- | --- |
|  | **Total (n=91)** | **Not ill (n=12/6**†**)** | **Mildly ill (n=58/31**†**)** | **Moderately ill (n=21/10**†**)** |
| **D0, n (%)** | 87 (95.6) | 10 (83.3) | 56 (96.6) | 21 (100) |
| **D7, n (%)** | 89 (97.8) | 12 (100) | 57 (98.3) | 20 (95.2) |
| **D14, n (%)** | 88 (96.7) | 11 (91.7) | 56 (96.6) | 21 (100) |
| **D28, n (%)** | 82 (90.1) | 10 (83.3) | 54 (93.1) | 20 (95.2) |
| **D42, n (%)** | 83 (91.2) | 11 (91.7) | 54 (93.1) | 18 (85.7) |
| **D180**† **(N=47), n (%)** | 47 (100) | 6 (100) | 31 (100) | 10 (100) |

†Only samples from unvaccinated individuals were included.
